# Supplementary material for: The discovery of novel noncoding RNAs in 50 bacterial genomes
Source: Nucleic Acids Res. 2024 Apr 22;52(9):5152–65. doi: 10.1093/nar/gkae248 (PMC11109978; doi:10.1093/nar/gkae248)
Supplement: gkae248_Supplemental_Files [file gkae248_supplemental_files.zip › Narunsky 50 Genomes RNA Motifs Files.zip!/Publication/56-D_turgidum/Figures/LRC-56-3-NC_011661_1_IGR_23_532900_533066.sto.pdf]

LRC-56-3-NC_011661_1_IGR_23_532900_533066.sto.r2r

5´-AAUGGGGCGGGAUUU●UCCCGCYCY●●●A●UU●●AAAG●●●●UUU●●AAA●GG●U●UUGRAGAUR●U●A●●●●R○○○○○○○○○○UR●GAGAARAGUARAGGRGGA○○○○AUAAUAUGCAAAUU●GAUAAY●GGUUU●RUAYUAUUU●●UUUUACYUUAAGUUUAUAU●UU●UCUA
